# From Case Reports to Molecular Insight: Examining the Outcomes and Underlying Mechanisms of Squamous Cell Carcinoma in Breast Implant Patients—A Systematic Review

**DOI:** 10.3390/ijms25052872

**Published:** 2024-03-01

**Authors:** Alexandra Camicia, Jose A. Foppiani, Otakar Raska, Angelica Hernandez Alvarez, Daniela Lee, Iulianna C. Taritsa, Kirsten A. Schuster, Rou Wan, Sylva Neradová, Gavin J. Lin, Theodore C. Lee, Martin Molitor, Michal Zikan, Samuel J. Lin

**Affiliations:** 1 Faculty of Medicine and Surgery, Campus Bio-Medico University of Rome, 00128 Rome, Italy; alexandra.camicia@alcampus.it; 2 Division of Plastic Surgery, Beth Israel Deaconess Medical Center, Harvard Medical School, Boston, MA 02215, USA; jfoppian@bidmc.harvard.edu (J.A.F.); ahernan8@bidmc.harvard.edu (A.H.A.); dlee23@bidmc.harvard.edu (D.L.); itarits1@bidmc.harvard.edu (I.C.T.); kaschust@bidmc.harvard.edu (K.A.S.); sjlin@bidmc.harvard.edu (S.J.L.); 3 Institute of Pathological Physiology, First Faculty of Medicine, Charles University, 12108 Praha, Czech Republic; snerads@gmail.com (S.N.); martin.molitor@bulovka.cz (M.M.); michal.zikan@lf1.cuni.cz (M.Z.); 4 Mayo Clinic, Rochester, MN 55902, USA; wan.rou@mayo.edu; 5 Nobles and Greenough School, Dedham, MA 02026, USA; 6 Georgetown University, Washington, DC 20001, USA; 7 Department of Plastic Surgery, Bulovka University Hospital, 46401 Praha, Czech Republic; 8 Department of Obstetrics and Gynecology, Bulovka University Hospital, 46401 Praha, Czech Republic

**Keywords:** BIA-SCC, clinical cases, molecular pathways

## Abstract

There is extensive coverage in the existing literature on implant-associated lymphomas like anaplastic large-cell lymphoma, but breast implant-associated squamous cell carcinoma (BIA-SCC) has received limited scholarly attention since its first case in 1992. Thus, this study aims to conduct a qualitative synthesis focused on the underexplored association between breast implants and BIA-SCC. A systematic review was conducted utilizing the PubMed, Web of Science, and Cochrane databases to identify all currently reported cases of BIA-SCC. Additionally, a literature review was performed to identify potential biochemical mechanisms that could lead to BIA-SCC. Studies were vetted for quality using the NIH quality assessment tool. From an initial pool of 246 papers, 11 met the quality criteria for inclusion, examining a total of 14 patients aged between 40 and 81 years. BIA-SCC was found in a diverse range of implants, including those with smooth and textured surfaces, as well as those filled with saline and silicone. The condition notably manifested a proclivity for aggressive clinical progression, as evidenced by a mortality rate approximating 21.4% within a post-diagnostic interval of six months. Our literature review reveals that chronic inflammation, driven by various external factors such as pathogens and implants, can initiate carcinogenesis through epigenetic modifications and immune system alterations. This includes effects from exosomes and macrophage polarization, showcasing potential pathways for the pathogenesis of BIA-SCC. The study highlights the pressing need for further investigation into BIA-SCC, a subject hitherto inadequately addressed in the academic sphere. This necessitates the urgency for early screening and intervention to improve postoperative outcomes. While the review is confined by its reliance on case reports and series, it serves as a valuable reference for future research endeavors.

## 1. Introduction

On 8 March 2023, the United States Food and Drug Administration (FDA) issued a safety alert, emphasizing the potential risk of squamous cell carcinoma (SCC) following breast implant surgery [1,2]. When the body responds to foreign objects like breast implants, the immune system attempts to isolate the implant by forming a collagenous capsule around it [3,4,5,6]. Within this encapsulated environment, SCC has been identified in a small subset of patients [7]. Cases of SCC localized within these fibrous capsules have been increasingly documented via case reports and series, with initial incidences traced back to the 1990s [8]. Although breast implant-associated squamous cell carcinoma (BIA-SCC) remains relatively rare, its potential for aggressiveness necessitates a rigorous examination of implant-related risks [7]. Therefore, it becomes imperative to provide patients with a comprehensive understanding of the range of risks they might face.

The existing academic literature on the topic has largely focused on the relationship between breast implants and various lymphomas, most notably breast implant-associated anaplastic large-cell lymphoma (BIA-ALCL) [9,10,11,12]. The exploration into the link between textured breast implants and the development of BIA-ALCL presents an intriguing look into how foreign body implantation can lead to lymphoma [9,10,11,12]. Despite our early stage of understanding, some theories have emerged from existing research, particularly focusing on the role of chronic inflammatory states in setting the stage for BIA-ALCL [9,10,11,12]. It is suggested that the texture of breast implants is a significant factor in the development of BIA-ALCL [9,10,11,12]. A prevailing theory suggests a connection between the chronic inflammatory state triggered by textured implants and the onset of BIA-ALCL [9,10,11,12]. The precise mechanisms connecting textured breast implants to BIA-ALCL are yet to be fully uncovered, calling for more detailed research to unravel the complex interaction of factors at the cellular and molecular levels. Additionally, the variability in disease presentation, from mild cases appearing as late periprosthetic fluid (seroma) to more severe lymphoma forms, highlights the complexity involved in the pathophysiology of BIA-ALCL [9,10,11,12]. In contrast, the possible connection between breast implants and the development of SCC has garnered comparatively limited empirical attention [8]. This area of inquiry is only recently acquiring prominence, underscoring the need for further research.

Similarly to BIA-ALCL, imaging techniques in BIA-SCC diagnosis often reveal fluid collection or tumor masses around the breast implant, while histopathological confirmation typically follows a biopsy or complete surgical removal, such as capsulectomy, to analyze the affected tissue. This approach ensures accurate diagnosis and informs the subsequent treatment strategy [13]. Unfortunately, unlike its BIA-ALCL counterpart, BIA-SCC tends to be more aggressive, and as such, its diagnosis is more pressing. The diagnostic process could be significantly enhanced by incorporating advanced technologies, such as automated computer-assisted decision-making systems. These systems are capable of detecting subtle asymmetries and other indicators that may suggest the presence of malignancy, thereby increasing the accuracy and speed of diagnosis [14].

At present, increasing evidence is substantiating the association between breast implants and SCC, although substantial gaps in current scientific knowledge still exist. In the event of a confirmed correlation, the responsibility lies in informing potential patients about these hidden risks and, when necessary, implementing effective screening protocols. Therefore, this qualitative systematic review aims to conduct a comprehensive analysis focused on the frequency, etiology, and clinical outcomes of SCC in patients with breast implants.

## 2. Methods

### 2.1. Systematic Review

This study protocol was prospectively registered with PROSPERO (Study#: CRD42023420842) [15]. Completion of the study was performed in accordance with the Preferred Reporting Items for Systematic Reviews and Meta-Analysis (PRISMA) statement guidelines [16].

#### 2.1.1. Eligibility Criteria

Criteria for included studies were defined as any studies reporting on the occurrence of BIA-SCC. The full eligibility criteria are reported in Table 1.

#### 2.1.2. Search Strategy

A comprehensive research review using subject headings, controlled vocabulary, and keywords was used to search on PubMed/MEDLINE and Web of Science for studies published until June 2023. Our full-text search strategy is accessible at PROSPERO.

#### 2.1.3. Study Selection

The search results were uploaded to Covidence. A two-stage screening process was conducted for study selection. Two screeners independently reviewed the titles and abstracts in the first step. In situations where disagreement arose between the initial two reviewers, a third reviewer was enlisted to decide on the ultimate inclusion or exclusion of the study, subsequent to a process of moderation. In the second stage, the same two reviewers performed a full-text review and selected studies that fulfilled the eligibility criteria. In situations where disagreement arose between the initial two reviewers, a third reviewer was enlisted to decide on the ultimate inclusion or exclusion of the study, subsequent to a process of moderation.

#### 2.1.4. Data Extraction/Synthesis

The variables extracted were patient characteristics, type of implants, therapy for the treatment of malignancy (chemotherapy, radiation), and outcomes.

#### 2.1.5. Quality Assessment

To assess the risk of bias, the National Institute of Health (NIH) quality assessment tool was utilized. Each article was categorized as follows: “low risk”, “moderate risk”, or “high risk” of bias [17].

#### 2.1.6. Statistical Analysis

Due to the heterogeneity of the topics covered in the studies constituting this systematic review, it was not possible to perform any analysis beyond a qualitative synthesis.

### 2.2. Literature Review

#### 2.2.1. Search Strategy

A comprehensive research review using subject headings, controlled vocabulary, and keywords was used to search on PubMed/MEDLINE and Web of Science for studies published until December 2023.

#### 2.2.2. Study Selection

Relevant studies highlighting the relationship between molecular pathways of chronic inflammation and carcinogenesis were selected.

#### 2.2.3. Data Extraction/Synthesis

A narrative synthesis was then performed to serve as a preliminary framework for understanding the molecular mechanisms potentially leading to BIA-SCC.

## 3. Results

### 3.1. Systematic Review

**Figure 1:**
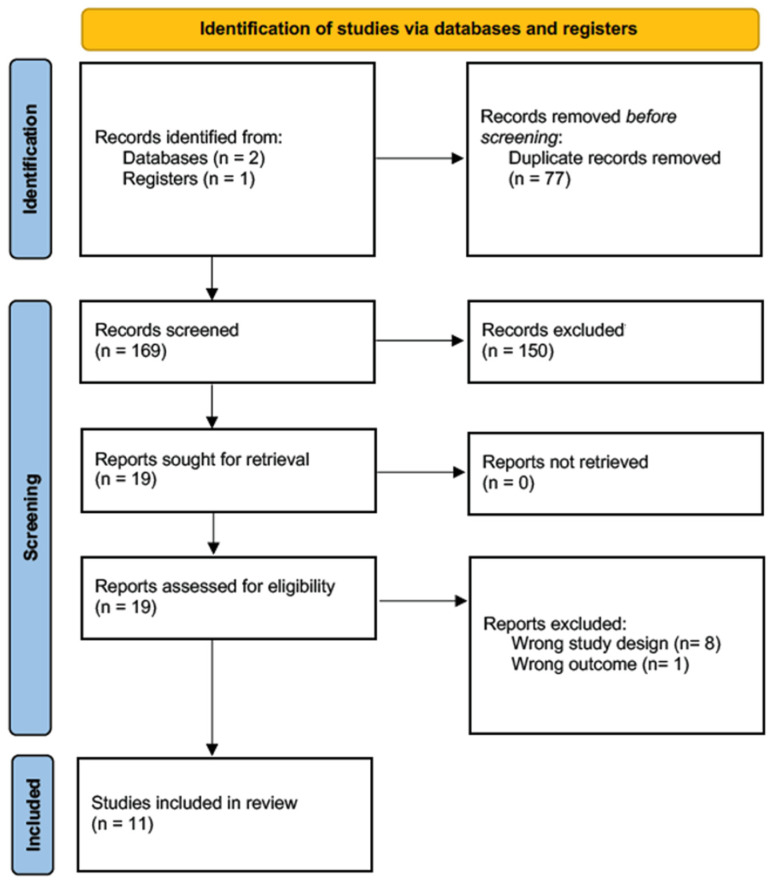
Prisma flow chart.

A total of 246 articles were initially identified, from which duplicates were subsequently removed. A set of 19 articles was subjected to a full-text review, and 11 were eventually selected for data extraction [7,18,19,20,21,22,23,24,25,26,27]. These articles specifically focused on the relationship between breast implants and SCC. Utilizing the NIH quality assessment tool, two of these articles were classified as “good”, five as “fair”, and four as “poor”. See Figure 1 and Table 1.

The studies cumulatively reported on 14 patients who were diagnosed with SCC post-breast implant insertion. The age at diagnosis ranged from 40 to 81 years, with the follow-up periods extending from one month to eight years. Importantly, the latency period from implant insertion to diagnosis for all patients exceeded 11 years, with a range of 11 to 42 years. See Table 2.

Among the patients, three had no recorded personal or family history of cancer. One patient had a history of metastatic colon cancer, one had a history of invasive ductal carcinoma status post-mastectomy, and one had a history of right breast carcinoma. For the remaining nine patients, this information was not available. Ten patients had undergone implantation for the purpose of cosmetic breast augmentation, and four patients had undergone implantation due to reconstruction. Typical clinical presentations of the BIA-SCC in our cohort included breast swelling, erythema, enlargement, and localized pain. See Table 3.

Out of the 14 patients, 3 had expired at the time of the last follow-up. Of these three, one individual exhibited distant metastases to the liver, lungs, and retroperitoneum. The second patient developed distant metastases within five months of the diagnosis. The third patient had been given palliative care one month into follow-up. Of the eleven remaining patients, six patients were reported as disease-free, one patient was in remission at 12 months after the initiation of adjuvant therapy, and one was alive but with ongoing disease (AWD). Follow-up information for the remaining three patients was unavailable.

As for implant types, 10 of the 14 patients initially received silicone implants, while the remaining 4 had saline implants. The majority of patients were treated with radical mastectomy, while the use of neoadjuvant or postoperative chemotherapy, chemoradiation, or radiotherapy was poorly recorded in the included studies. See Table 4. 

### 3.2. Literature Review

#### 3.2.1. Inflammation and Carcinogenesis

Chronic inflammation is increasingly recognized as a significant catalyst in the development of various cancer types. The relationship between chronic inflammation and cancer development posits a complex interplay of cytokines, growth factors, and immune cells, creating an environment conducive to tumorigenesis [28,29,30]. This section delves into the underlying mechanisms of this relationship, laying the groundwork for understanding specific cancer types, including BIA-SCC.

Research by Xiong et al. and Nicolò et al. underscores the role of chronic inflammation in eliciting cancer [31,32]. They highlight how long-term exposure to inflammatory stimuli, such as pathogenic infections and persistent immune response, can lead to genetic mutations and cellular transformations. This process is a critical element in the development of lung cancer, as demonstrated by Xiong et al., where *Mycobacterium tuberculosis* infection leads to chronic inflammation, significantly contributing to lung cancer progression [31]. Similarly, Nicolò et al. discuss how bacterial infections, distinct from Lactobacillus, can induce a chronic inflammatory state, facilitating the neoplastic progression in human papillomavirus (HPV)-infected epithelial cells [32].

#### 3.2.2. Breast Implant Material

Historically, breast implants utilized problematic materials like polyurethane and Teflon, leading to various complications. Saline-filled implants, though minimally invasive, failed to mimic the natural feel of breast tissue [33]. Innovations introduced smooth silicone elastomer shells to replicate the breast’s anatomy, yet faced challenges like displacement and capsular contracture, prompting the use of Dacron patches and textured surfaces to address these issues [33]. However, textured implants brought new problems, such as seroma and BIA-ALCL. Modern silicone-based implants feature a gel filler and elastomer shell, designed with varying viscosities and textures to optimize outcomes. Continuous advancements aim to refine implant surfaces, balancing technological improvements with persistent challenges [33].

#### 3.2.3. Chronic Inflammation: A Catalyst for Tumorigenesis

Chronic inflammation can drive tumorigenesis through various mechanisms, primarily involving immune cell recruitment and the release of pro-inflammatory cytokines [28,29,30]. Gubernatorova et al. emphasize the dual role of tumor necrosis factor (TNF) and lymphotoxin (LTα) in cancer. In their study, different mouse models showed that TNF can drive lung metastasis, providing another example of inflammation-induced carcinogenesis [34,35].

Other specialized immune cells have been implicated in the connection between inflammation and cancer, including macrophages [34]. The endocytosis of mycobacterium tuberculosis by alveolar macrophages and the subsequent downstream immune response have been shown to cause sustained inflammation and tissue damage, driving cancer development [31]. T cells also play a central role in the inflammatory milieu. Exhausted T cell phenotypes, enriched in granulomas, can contribute to the development of tumors. This is further affected by the expression of immune checkpoints like PD-1 and LAG-3 in these T cells, which inhibit effective immune response and facilitate tumor progression [35].

These studies collectively underscore the critical role of chronic inflammation in cancer. The recruitment of immune cells, combined with the sustained release of inflammatory cytokines and growth factors, creates an environment that promotes the proliferation and survival of cancer cells [28]. This understanding provides a crucial foundation for examining specific cancer types, particularly BIA-SCC, in the context of inflammation-induced carcinogenesis.

#### 3.2.4. Inflammatory Mediators and Tumor Microenvironment

The tumor microenvironment (TME), heavily influenced by inflammatory mediators, plays a pivotal role in cancer progression [35,36]. Chronic inflammation within the TME facilitates cellular transformations leading to cancer. It has been demonstrated that macrophage-secreted cytokines like tumor necrosis factor (TNF) and interleukins activate cancer-associated pathways such as NF-κB in pulmonary epithelial cells. LTα in the TME has also been shown to promote tumor growth and metastasis [28]. This highlights the complexity of the inflammatory response in the TME and its impact on cancer development [35,36]. Apart from cytokines, exosomes derived from macrophage-processed cancer cells may play a role in the TME. As discussed in the work of Xiang et al., breast cancer cell-derived exosomes can induce macrophages into an M2 polarization state, which is typically associated with tissue repair and tumor progression [36]. These exosomes alter the TME, promoting conditions favorable for cancer growth [35,36].

#### 3.2.5. Pathways Leading to BIA-SCC

In the context of BIA-SCC, the chronic inflammatory response to breast implants may serve as a critical factor in carcinogenesis. Doloff et al. demonstrate that different surface topographies of breast implants can induce varying immune responses, potentially leading to fibrosis and other chronic inflammatory states [37]. Their study shows that smoother implant surfaces can reduce fibrosis and immune cell recruitment, suggesting that implant surface characteristics significantly influence the local immune response.

The research by Ghosh et al. further supports this notion. Their immunopeptidomic analysis reveals that biomaterial contact can alter the immune system’s response, showing potential pathways for immune system dysregulation leading to cancer [38]. Materials used in breast implants might induce specific immune responses that could contribute to the development of BIA-SCC.

Furthermore, the studies by Xiong et al., Nicolò et al., and Gubernatorova et al. provide insights into how chronic inflammation and immune dysregulation, potentially caused by breast implants, can lead to carcinogenesis [31,32,35]. The prolonged inflammatory state, combined with the release of cytokines and growth factors, and changes in the immune cell profiles within the TME, may create conditions that promote the development of BIA-SCC.

#### 3.2.6. Chronic Inflammation and Epigenetic Changes in Cancer

Chronic inflammation is known to induce epigenetic changes that can lead to cancer. The studies by Xiong et al. and Nicolò et al. highlight how chronic inflammatory conditions, such as those caused by infections or the presence of foreign bodies, can lead to alterations in DNA methylation and histone modification [31,32]. These epigenetic changes are crucial in the transition from a normal cell to a cancer cell. The epigenetic changes associated with chronic inflammation have been shown to be implicated in disease progression in breast cancer and cervical intraepithelial neoplasia in the context of bacterial or viral infection [32]. These mechanisms can be extrapolated to understand the development of BIA-SCC, where chronic inflammation around the implant site could lead to epigenetic modifications in the surrounding breast tissue, predisposing it to malignant transformation.

#### 3.2.7. The Role of Exosomes and Macrophage Polarization in Tumor Progression

The studies by Xiang et al. and Doloff et al. provide insights into the role of exosomes and macrophage polarization in tumor progression [36,37]. Exosomes driving breast cancer progression, as shown by Xiang et al. in the setting of chronic infection, may also have a role in the setting of patients with breast implants [36]. Here, chronic inflammation or the implant material itself could alter the local immune response, promoting exosome production conducive to cancer development.

Doloff et al. further elucidate how different implant surface topographies can modulate the immune response, including the behavior of macrophages [37]. They demonstrate that smoother implant surfaces can lead to a reduced fibrotic and inflammatory response, suggesting that the physical characteristics of breast implants can significantly influence the local immune environment. This could have implications for the development of BIA-SCC, as a pro-inflammatory and fibrotic microenvironment around rougher implant surfaces might facilitate the transformation of adjacent breast tissue into squamous cell carcinoma.

These studies collectively suggest a complex interplay of factors in the development of BIA-SCC, involving chronic inflammation, exosome-mediated communication, macrophage polarization, and the physical properties of breast implants. Understanding these mechanisms is essential for improving the design and safety of breast implants to reduce the risk of BIA-SCC.

## 4. Discussion

Breast implantation, a procedure conducted for both reconstructive and esthetic enhancement of the breast, is not without its associated risks and complications [39,40]. While existing research has shed light on the relationship between breast implants and ALCL, the intersection between breast implants and SCC is relatively underexplored [41]. This dearth of data is even more significant given that SCC usually emerges around the implant capsule and appears to affect both smooth and textured as well as both silicone and saline implants.

Given the limited understanding of the pathophysiology of BIA-SCC, it is reasonable to consider chronic inflammation as a plausible underpinning [41,42,43]. Indeed, chronic inflammation has been implicated in the development of various types of cancers, and the literature on the topic posits that SCC may be mediated by sustained inflammatory processes [41,42,43,44,45,46]. Notably, inflammation could arise from mechanical factors, such as implant movement, or, even less likely, microbiological factors [41,42,43,44,45,46]. Chronic inflammation can exacerbate the production of reactive oxygen species and other pro-inflammatory cytokines, creating an environment conducive to neoplastic transformations. In other malignancies associated with breast implants like BIA-ALCL, hypotheses have already been advanced regarding the role of inflammation [46]. Indeed, BIA-ALCL disease emerges in an inflammatory microenvironment rich in lymphocyte and plasma cell infiltration, showing a pronounced Th1/Th17 phenotype in advanced stages [41,42,43,46]. Genetic alterations affecting the JAK/STAT signaling pathway are also commonly observed. This possibly provides a template for understanding BIA-SCC [41,42,43].

As we navigate further into this intricate web of potential associations between breast implants and squamous cell carcinoma, it is prudent to recognize the etiological complexity of this pathology. Both genetic predisposition and environmental triggers may synergistically contribute to the genesis of BIA-SCC, implicating a multifactorial causation model. Adding a layer of complexity is the finding that BIA-SCC is not entirely specific to any particular type of implant. This suggests that the mere presence of a foreign body within the breast may itself act as a catalyst, instigating aberrant cell proliferation irrespective of the implant’s material or textural composition. This is notably different from BIA-ALCL, which has a strong association with macro-textured implants and tissue expanders [47]. Such a supposition further intensifies the need for a multifaceted risk-assessment strategy that transcends the physical properties of the implant to encapsulate a broader range of biological, mechanical, and environmental factors.

It is noteworthy that the onset of SCC often manifests more than a decade post-implantation with a high degree of clinical heterogeneity as seen in our cohort [7,18,19,20,21,22,23,24,25,26,27]. While most patients present with swelling and pain, these symptoms are non-specific and can easily be attributed to more benign conditions, thereby introducing the risk of diagnostic inertia [7,18,19,20,21,22,23,24,25,26,27]. Therefore, heightened clinical acumen, supplemented by advanced diagnostic modalities, is integral to ensuring early detection and intervention. Furthermore, our cohort exhibited a distinctively aggressive clinical course, with a high mortality rate and resistance to conventional therapies like chemotherapy and radiotherapy [7,18,19,20,21,22,23,24,25,26,27]. Despite the aggressive course of BIA-SCC, en bloc capsulectomy appears to yield superior outcomes, highlighting the importance of complete surgical resection [7,18,19,20,21,22,23,24,25,26,27].

The ASPS/PSF offers limited recommendations that include preoperative workups and particular surgical protocols designed to facilitate the diagnosis and management of BIA-SCC and other lymphomas [1,2]. Consequently, there is a pronounced need for the assembly of an expert panel to standardize treatment protocols, ensuring data collection and effective patient management. Continuous patient education and vigilant, long-term monitoring are also pivotal in optimizing patient outcomes, given the prolonged indolent nature of BIA-SCC and its often-late presentation.

While mortality rates in our cohort were indeed alarming, it is crucial to realize that these statistics may not be universally generalizable due to the restricted scope and size of our study sample. Therefore, these findings should act as an impetus for more expansive research initiatives, incorporating a wider demographic and clinical spectrum to facilitate a more nuanced understanding of BIA-SCC. The establishment of dedicated sub-registries for BIA-SCC would serve as invaluable repositories of clinical data, aiding in the formulation of more evidence-based diagnostic and therapeutic algorithms.

## 5. Limitations

While this paper serves as a comprehensive compilation of available data on BIA-SCC, its intrinsic limitations lie in the nature of the included studies, which are mostly case reports and series. Therefore, further robust, multicentric research is essential to bridge the existing knowledge gap and to refine the clinical guidelines for the effective management of BIA-SCC. We must exercise caution when extrapolating the pathogenetic mechanisms identified in other malignancies and SCC instances to BIA-SCC, as discussed in this review. These mechanisms are presented as theoretical possibilities, offering insight into potential biological pathways that could be relevant to BIA-SCC’s pathogenesis.

## 6. Conclusions

In conclusion, our understanding of BIA-SCC remains in its nascent stages. It is an amalgam of incomplete data, emerging theories, and clinical observations that warrant rigorous scientific investigation. By adopting a methodical, multidisciplinary approach that integrates advancements in molecular biology, surgical techniques, and patient care protocols, we have the potential to unravel the complexities of BIA-SCC. These collective efforts are not only academic endeavors but also ethical imperatives, with the aim of protecting the wellbeing of the numerous individuals who opt for breast implantation each year.

## Figures and Tables

**Table 1:** Eligibility criteria.

Inclusion Criteria	Exclusion Criteria
Published before 23 May 2023	Published after 23 May 2023
Patients older than 18 years old	Patients younger than 18 years old
Observational studies, randomized controlled trials	Systematic review
Case series, case reports	Literature review
Written in English	Case cadaver studies, animal studies
Breast implant-associated squamous cell carcinoma	

**Table 2:** Study characteristics.

Title	Author Name, Year	Type Of Implant	Number of Patients	NIH Quality Assessment
Squamous Cell Carcinoma Arising In Breast Implant Capsules	Goldberg et al., 2021 [18]	Patient (1) Smooth round saline sub-muscular 350 mL implants; Patient (2) Smooth silicone implants without use of autologous flaps	2	Good
Squamous Cell Carcinoma Following Breast Augmentation	Paletta et al., 1992 [19]	Silicone implant (Heyer-Schulte 240 mL style 2100 silicone gel mammary protheses)	1	Poor
Epithelialization Of The Lining Of A Breast Implant Capsule. Possible Origins Of SCC associated with a breast implant capsule	Kitchen et al., 1994 [20]	Patient (1) Silicone implant; Patient (2) 240 mL style 2100 Heyer-Schulte silicone gel protheses	1	Poor
Breast Implant Capsule-Associated SCC: a report of 2 cases	Olsen et al., 2017 [21]	Patient (1) In 1984, she had silicone breast implants, and after contracture, they were replaced with 300 mL textured saline implant; Patient (2) Unknown silicone implant	2	Good
Primary SCC Arising From A Breast Implant Capsule	Zomerlei et al., 2015 [22]	Smooth silicone implant; she then required multiple subsequent procedures (due to right-sided capsular contracture), exchange to saline implants, then back to smooth silicone implants	1	Fair
Primary SCC Arising From A Breast Implant Capsule: A case report and review of the literature	Buchanan et al., 2018 [23]	Foam-covered silastic (Heyer-Schulte 200 cc foam-covered silastic implants)	1	Fair
Breast Implant Capsule-Associated SCC during Pregnancy: A Mimicker of Breast Implant Associated Anaplastic Large Cell Lymphoma	Soni et al., 2022. [24]	Smooth round saline submuscular implants and two previous revisions for capsular contracture	1	Poor
Breast Implant Capsule-Associated SCC: report of 2 patients	Whaley et al., 2022 [7]	Patient (1) Textured saline implants Left 340 cc, Right 240 cc—Manufacture designation “MCGH”; Patient (2) Bilateral saline implants	2	Fair
Breast Squamous Cell Carcinoma Following Breast Augmentation	Zhou et al., 2018 [25]	Silicone gel implants	1	Fair
Squamous metaplasia on the breast implant capsule	Alikhan et al., 2010 [26]	Silicone implants	1	Fair
Breast implant-associated squamous cell carcinoma—a rare long term complication	Satgunaseelan et al., 2015 [27]	Not reported	1	Poor

**Table 3:** Patient characteristics.

Author Name, Year	Number of Patients	Age at Diagnosis	Purpose of Surgery (Cosmetic, Reconstructive)	Time until Diagnosis	History of Cancer
Goldberg et al., 2021 [18]	2	Patient (1) 40 y; Patient (2) 62 y	Patient (1) Cosmetic; Patient (2) Reconstruction (post-benign lesion excision)	Patient (1) 11 y; Patient (2) 32 y	No personal or family history
Paletta et al., 1992 [19]	1	52 y	Cosmetic (bilateral breast augmentation)	16 y	Not reported
Kitchen et al., 1994 [20]	1	52 y	Cosmetic	25 y	Not reported
Olsen et al., 2017 [21]	2	Patient (1) 56 y; Patient (2) 81 y	Patient (1) Cosmetic; Patient (2) Reconstruction (post-benign lesion excision)	Patient (1) 18 y; Patient (2) 42 y	Not reported (both patients)
Zomerlei et al., 2015 [22]	1	58 y	Cosmetic (bilateral augmentation mammoplasty)	15 y	Not reported
Buchanan et al., 2018 [23]	1	65 y	Cosmetic	35 y	Not reported
Soni et al., 2022 [24]	1	46 y	Cosmetic	Not reported	Not reported
Whaley et al., 2022 [7]	2	Patient (1) 60 y; Patient (2) 57 y	Cosmetic	Patient (1) 26 y; Patient (2); 25 y	Patient (1) None; Patient (2) Metastatic colon cancer
Zhou et al., 2018 [25]	1	46 y	Cosmetic	23 y	Not reported
Alikhan et al., 2010 [26]	1	70 y	Reconstruction	16 y	She had a history of right breast carcinoma
Satgunaseelan et al., 2015 [27]	1	58 y	Reconstruction	29 y	Not reported

**Table 4:** Patient presentation and treatment.

Author Name, Year	Number of Patients	Presentation	Follow-Up	Therapeutic Treatment
Goldberg et al., 2021 [18]	2	Patient (1) Sudden onset of swelling and erythema of the left breast 10 days after sustaining blunt trauma to her chest; discharge from nipple at injury; Patient (2) Right breast swelling and pain for 2 months after falling on her chest	Patient (1) Expired from malignant pleural effusions at 3-month follow-up; Patient (2) Lost to follow-up	Patient (1) Neoadjuvant chemotherapy, patient expired before chest wall resection; Patient (2) Chemoradiation
Paletta et al., 1992 [19]	1	Painful, enlarged left breast. She stated her left breast gradually began to increase in size 4 weeks before presentation. She denied nipple discharge, breast trauma, or recent systemic infections.	Disease-free at 12-month follow-up	Radical mastectomy
Kitchen et al., 1994 [20]	1	Patient (1) Intermittent pain in both breasts presented 1 year ago and exacerbated during activity and exercise; Patient (2) Left capsular contracture and enlarged (twice as large as the right breast) and painful left breast for 4 weeks	Not reported	Modified radical mastectomy
Olsen et al., 2017 [21]	2	Patient (1) 4-week history of painful, enlarged left breast with associated red-purple discoloration; Patient (2) Palpable left breast mass	Patient (1) Palliative care at 1-month follow-up; Patient (2) Distant metastases at 5-month follow-up	Patient (1) Mastectomy with postoperative chemotherapy; Patient (2) Mastectomy
Zomerlei et al., 2015 [22]	1	Sudden onset of right breast pain, swelling, and erythema; 2–3× enlargement of right breast relative to the left and thinning of the overlying skin	Not reported	Right total mastectomy, complete capsulectomy with concurrent left explant and simple mastectomy (per patient request)
Buchanan et al., 2018 [23]	1	Enlarged left breast after a mechanical fall after slipping while at home; left breast approximately 2× the size of the right and extremely tender to palpation	Disease-free at 8-year follow-up	Radical mastectomy and adjuvant radiation
Soni et al., 2022 [24]	1	4-month history of pain and swelling in the right breast	In remission 12 months after initiation of adjuvant therapy	Adjuvant chemotherapy and radiotherapy
Whaley et al., 2022 [7]	2	Patient (1) Left breast pain, significant swelling (tripled in size), and purulent drainage; Patient (2) Capsular contracture, pain, and swelling	Patient (1) NED; Patient (2) AWD	Patient (1) Patient underwent surgery for bilateral breast implant removal and capsulectomy—did not receive radiation or chemotherapy; Patient (2) Capsulectomy and excision of wound edges
Zhou et al., 2018 [25]	1	Hardening and swelling of her right breast	Follow-up within a year showed metastasis to liver, lungs, and retroperitoneum. She expired of her disease in July 2017	External beam radiation
Alikhan et al., 2010 [26]	1	Change in shape and size of her right breast	After capsulectomy and implant exchange, no abnormalities were reported by the surgeon	Capsulectomy
Satgunaseelan et al., 2015 [27]	1	Pain and induration of her left breast, following the third surgical revision of her breast implants initially inserted in 1985	No evidence of residual malignancy	Completion mastectomy

## Data Availability

No new data were created or analyzed in this study. Data sharing is not applicable to this article. All information relevant to this systematic review is part of the manuscript, figures, tables, and/or digital supplemental content. Additional information can be found within the publicly available PROSPERO protocol for this study. If any further information is required, the reader may contact the corresponding author for clarifications.
